# 

**Published:** 1887-05-14

**Authors:** 


### The Hospital.

**An Institution, Family, and Parochial Journal of Hospitals, Asylums, and all Agencies for the Care of the Sick, Criticism and News.**

SATURDAY, MAY 14TH, 1887.

### NOTICE TO CORRESPONDENTS.

Mr. Charles Harrison is thanked for his communication and enclosures.

### Notice to Advertisers.

*To insure prompt insertion, all Advertisements for* THE HOSPITAL *must be sent in direct to the Advertising Agent, Mr. A. P. Watt, 2, Paternoster Square, E.C.*

### The Literary Prize.

A PRIZE of **Two Guineas** will be given every month for the best story or incident based upon hospital, or asylum, or student life, or any incident connected with the professions of medicine or nursing.

### QUESTIONS AND ANSWERS.

All questions should be addressed to Mr. Howard J. Collins, Secretary, The Hospitals Association, Norfolk House, Norfolk Street, Strand, W.C.

### THE QUILTER PRIZE HOSPITAL ACCOUNT FORMS.

With reference to the form of account sent out to the Quilter Prize competitors, and the desire expressed that it might be universally adopted in hospitals, Mr. Thomas Robinson, the Secretary of the Sunderland Infirmary, writes: "I have had the pleasure of submitting your letter of March 15th to our general committee, and I am directed to say that they will consent to have their accounts analysed as suggested by you, providing a fair proportion of the hospitals are likely to adopt the form, so that comparisons may easily be made. I shall be glad to learn at your convenience what prospect there is of the form being generally adopted?"

*** We are glad to say that an increasing number of hospitals are adopting the form referred to, and the prospect of securing uniformity of account-keeping in hospitals and kindred institutions is improving. The question, however, is surrounded with difficulties, and prejudice, as well as the increased trouble which an alteration in the system of accounts entails, combine to delay the universal adoption of an uniform system.

### THE CHILDREN'S CORNER.

**Third Quarterly Prize Competition.**
Began 2nd Apr., 1887 ; ends 25th June, 1887.

**Puzzles—Seventh Week.**

No. 27. CONUNDRUM. In what way does a hen sitting on a barn door resemble a halfpenny ?

No. 28. NAMES OF TOWNS ENIGMATICALLY EXPRESSED. Part of a ship ; to arouse and a piece of land generally enclosed by a hedge ; a small nail and a shallow part of a river ; a Puritan poet ; to divide and a body of earth.

No. 29. ENIGMA.

" On, Stanley, on !" were the last words of Marmion.
If I had been in Stanley's place,
When Marmion urged him to the chase,
I there a thing should then descry,
Would bring a tear to many an eye.

No. 30. CHARADE.

In my *first*, my *second* sat,
My *third* and *fourth* I ate,
Can you now just tell me that
Which I here indicate ?

**Letters—Seventh Week.**

The Letter-subject for next week will be "Reading."

**Results of Fifth Week—Puzzles.**

No. 20. CONUNDRUM. Because it is a fowl (foul) proceeding towards Parliament.

No. 21. ENIGMA. First he takes the goose over. He returns and fetches the fox ; recrosses, taking the goose ; returns again, carrying the barley ; then he finally goes back and brings over the goose.

No. 22. CHARADE. The last line reveals the answer, "Thou tea-chest art"—Tea chest.

No. 23. BURIED AUTHORS. Shakespeare, Hood, Burns, Johnson (or Thompson, Richardson, etc.), More, Locke.

*All right*—Peeler, A. G. S. McCulloch, Dot, Mikado, Bohemian Girl, Umslopogaas, Tim, Joe, A C. K., Gom, Sutton, Nordisa ; *Three right*—R. Epton, Skye, Violin, The Eda, Scrooge, Alsie ; *Two right*—Agatha, Ellie, Etta.

**Letters.**

I have had some very pretty Country Rambles described to me—walks so lovely that I only wish I could have accompanied some of my young friends in them. Violin writes me her first lettter, and an admirable letter it is, save that she breaks one of my rules by writing on both sides of the paper. Ellie nicely describes an early morning ramble before breakfast. Here is a very characteristic note from Mikado :—

DEAR MR. EDITOR,—My idea of a "Country Ramble" is to start about nine o'clock in the morning, and walk for six or seven miles till you come to a nice wood ; then to have the lunch you have brought with you, rest a little while, and afterwards wander about in search of ferns or flowers to fill the basket, and about six o'clock to go to the nearest village for new milk and a good tea. Then to walk home to supper in the dusk, or, if there happens to be a moon, in the moonlight. Is this such a day as you would like ? MIKADO, aged 15.

12, Upper Tichborne Street, Leicester.

Ada loves a country ramble which cheers the spirit, and, as she very rightly says, evokes in us a feeling of thankfulness to our Heavenly Father who has given us all these good things to enjoy. Skye's letters always remind me of the story of *Eyes and no Eyes*. How much some find to observe wheresoever they go, while others can see next to nothing. Skye is a little child who sees much and intelligently notes all she observes. Here is a letter from her :—

DEAR MR. EDITOR,—We are staying at Herne Bay for a week, and I went for a very nice walk yesterday to the little village of Herne. It is a very pleasant country walk over green fields, with a fine view of the sea for part of the way. The great attraction to the village is its ancient church, which was built in the thirteenth century. It used to be a Roman Catholic church, and there is some very interesting carving done by the old monks on some of the pews, and on a pulpit in which Ridley preached. He was vicar of Herne for nearly twelve years. I think it was from there that he was taken to be burnt. There is a helmet hanging up in the church of a knight who would hunt on Sundays, and was killed by his own dogs on a Sunday.

Yours faithfully, SKYE, aged 14.

4, Milton Terrace, Chatham.

Agatha tells me about a beautiful ramble to Horner Combe, a kind of valley in Somerset. Etta writes very nicely, and her letter afforded me much interest. Peeler and Bohemian Girl both write excellent letters.

One mark each:—Bohemian Girl, Ellie, Mikado, Peeler, Skye, Agatha, Ada, Etta, Violin.

Answers must be addressed to the "PUZZLE EDITOR," THE HOSPITAL, Norfolk House, Norfolk Street, Strand, W.C., not later than Thursday next. The "Children's Corner" Coupon (see advertisement pages) must be enclosed.

### AMUSEMENTS WITH PROFIT.

### PRIZE COMPETITIONS.

**Quarterly Prize Competition.**
Began 23rd April ; ends 16th July.

**A Prize of Three Guineas**

will be awarded to the Competitor who shall have sent in the largest number of correct or best answers. No one will be permitted to win the Three Guinea Prize twice in any one year, but we shall award annually an additional

**Prize of Five Guineas**

to the competitor who shall have sent in the largest number of correct or best answers during the twelve months.

**No. 7. Mathematical Question.**

It is required by means of four weights to weigh every number of lbs. from 1 up to 40 inclusive. The four weights are together to be equivalent to 40 lb. What should the weights be ?

**No. 8. Charade.**

My *first* is spread over my *second*,
And then my whole perfect is reckon'd,
But I'll tell you more clearly—now, guess if you can,
From his birth to his death, I'm the history of man.

**Answers.**

No. 3. DOUBLE ACROSTIC.

B ac H
E ssiquib O
L ycurgu S
G ossi P
R ossett I
A los T
V ienn A
E nglish Channe L

Correct answers have been received from Ellice, Gip, The Man of the Sea, Dawnlover, Gretta, Mater, Aralc, Broadwater, K. M., Letheädor, Peeler, Canice, Ostrich, Dolphin, Robin, May, A. M. E., Common Sense, Mrs. Bedingfeld.

No. 4. MATHEMATICAL QUESTION.

Each period of four years = 1461 days.

$$\text{Now, } \frac{30,000}{1,461} = 20 \text{ periods of 4 years} + 780 \text{ days} = 80 \text{ years} + 780 \text{ days.}$$

This calculation takes us back to June 21, 1771, but as the year 1800 was not a leap year (18 not being divisible by 4), we must clearly go back one day more in the calendar, viz., to June 20, 1771.

$$\text{Now, } \frac{780}{365} = 2 \text{ years, 50 days.}$$

Hence we get the date of the man's birth, May 1, 1769.

Correct answers have been received from Ellice, Dawnlover, Gretta, Mater, Broadwater, Letheädor, Ostrich, Grip, Boss.

**The Prize Tie.**

Ostrich and Ellice having elected to decide the prize by competition, we set the following question. No credit will, of course, be given to any other solvers who may answer it, nor will its correct solution (in the case of tied winners) count for the annual prize.

TEST ACROSTIC.

From great Apollo sprung, yet tried by human woe,
Saved from the fiery flame, yet by the same laid low,
First of all healers of disease in ancient days,
This was his worthy title, this his deathless praise.

From studying this, a modern *science* springs ;
This, whether man or reptile, to his subject *clings ;*
A science here, by which the *eye* can *penetrate ;*
The kingdom of the gods, 'midst whom *this holds his state ;*
This was the *haunt and theme* of sacred kings of old ;
This has the sound of *want*, but means a *mass of gold ;*
From this, in these *exacting times*, we would be free ;
The great *Pantheist* of the Dutch *he* claims to be ;
A judge of Israel's tribes, of mighty *strength* was he.

**Notices to Correspondents.**

DAWNLOVER.—Yes ; the Iron Duke is the man referred to in Question 4. His birthday is, as you say, somewhat disputed.

PERSEVERANCE.—Redcar for Ravenspur of course disqualified you for No. 25. Every light must be correct.

Answers must be addressed to THE PRIZE EDITOR, AMUSEMENTS WITH PROFIT, THE HOSPITAL, Norfolk House, Norfolk Street, Strand, W.C., not later than the *first* post on Saturday next. The full name and address must in all cases be given, but a *nom de plume* may be added for publication. Only one side of the paper should be written on. The "Amusements with Profit" Coupon (see advertisement pages) must be enclosed with replies.

### The In- and Out-Patients' Hospital Diary.

This Diary is so arranged as to make some portion of it appear every week, and the whole in each monthly part. It has been verv difficult to compile, and corrections will at all times be gratefully received. It has been suggested that similar information about the larger Provincial and Scotch Hospitals would be useful to many readers. We should be glad to hear if this is felt to be the case.

### CHILDREN'S DISEASES.—*continued.*

| Hospitals and Terms of Admission. | Physicians, Surgeons, and Medical Officers, with Days and Hours of Attendance. |
|---|---|
| Samaritan Free Hospital for Women and Children, Lower Seymour Street, W.; Branch, 1, Dorset St., W. Sec., Mr. G. Scudamore. In-patients must be between 2 and 12. Out-patients Department (free) is at Edward's Mews, Duke Street, W. | *In-Physician*, Dr. W. H. Day. Daily, 2 *In-Surgeon*, Mr. W. A. Meredith. Daily, 2 *Out-Physicians*, Drs. M. Prickett and Amand Routh, W. S. 1.30 *Out-Surgeons*, Messrs. W. A. Meredith and J. D. Malcolm, M. Th.; A. H. G. Doran and W. S. A. Griffith, Tu. F., 1.30 |
| Victoria Hosp. for Children, Queen's Road, Chelsea, S.W. Sec., Capt. W. C. Blount, R.N. Age of boys, 2 to 12 years; age of girls, 2 to 16. Admission for in-patients by subscriber's letter. Accidents and urgent cases free at all times | *In-Physicians*, Drs. J. Evans, M. Th. 2; Ridge-Jones, Tu. F. 2 *In-Surgeons*, Messrs. Pick, Tu. F. 9.15; Clutton, M. Th. 4.30 *Out-Physicians*, Drs. A. Venn, W. S. 9.30; C. Fox, M. Th. 1.30; F. D. Drewitt, Tu. F. 9; J. Philpot, M. Th. 9 *Out-Surgeons*, Messrs. F. Churchill, Tu. F. 10; W. Pye, M. Th. 9.30 |
| West London Hospital, Hammersmith rd, W. | Daily, 2. *See* General Hospitals |

### CONSUMPTION AND DISEASES OF THE CHEST.

| | |
|---|---|
| City of London Hospital for Diseases of the Chest, Victoria Park, E. Sec., Mr. T. Storrar Smith. By subscriber's letter available for 6 weeks. | *In-Physicians*, Drs. J. Thorowgood, S.; E. Smith, M.; J. Fothergill, W.; S. West, F.; G. A. Heron, W.; V. D. Harris, Tu. Hour, 2 *Out-Physicians*, Drs. V. D. Harris, Tu.; J. A. Ormerod, Th.; E. C. Beale, Tu. F.; B. Fenwick, W. S.; A. Money, W. S.; L. E. Shaw, M. Th. Hour, 2 |
| Hospital for Consumption, Fulham road, Brompton, S.W. Sec., Mr. H. Dobbin. Admission by letter of recommendation, but, where unobtainable, application may be made to the Secretary at the Hospital. | *In-Physicians*, Drs. E. S. Thompson, M. Th.; C. T. Williams, M. Th.; R. D. Powell, Tu. F.; J. Tatham, W. S.; R. E. Thompson, W. S.; F. T. Roberts, Tu. F. Hours, 2 to 4 *In-Surgeon*, Mr. R. J. Godlee, F. *Out-Physicians*, Drs. T. H. Green, J. M. Bruce, M. Th.; J. K. Fowler, W. S.; P. Kidd and C. Y. Biss, Tu. F.; T. D. Acland, W. S. Hour, 12 |
| North London Hospital for Consumption and Diseases of the Chest, Mount Vernon, Hampstead, N. Sec., Mr. L. F. Hill. Admission by subscriber's letter available for 6 weeks. | *In-Physicians*, Drs. A. Evershed, Tu.; B. O'Connor, F.; D. Pidcock, F. Hour, 10.30 *Out-Physicians*, Drs. B. O'Connor, D. Pidcock, J. E. Squire. Daily, 2 (attend at Out-patients' Branch, 216, Tottenham Court Road, W.) |
| Royal Hospital for Diseases of the Chest, 231, City Rd., E.C. Sec., Mr. John J. Austin. By Governor's letter, available for six weeks | *In-Physicians*, Drs. Hensley, W.; Gilbart-Smith, M.; Finlay, Th. *In-Surgeon*, Mr. A. Pearce-Gould, M. Tu. W. Th. F. 2, S. 9 *Out-Physicians*, Drs. White, Tu. F.; Pringle, M. Th.; O. Browne, W. S.; A. Davies, M. F.; H. Habershon, W. S.; E. Stewart, Tu. Th. Hour, 1; S. 9 |
| Royal National Hosp. for Consumption and Diseases of the Chest, Ventnor. Offices, 34, Craven St., Strand, W.C. Sec., Mr. E. Morgan. Admits only incipient cases by Governor's letter. | *In-and Out-Physicians*, Drs. J. G. Sinclair Coghill, and R. Robertson attend daily (at Ventnor) on out-patients at 11; on in-patients at noon *In- and Out-Surgeons*, Messrs. Whitehead, Williamson |

### EAR CASES.

| | |
|---|---|
| Central London Throat and Ear Hosp., Gray's Inn Rd., W.C. Sec., Mr. R. Kershaw. Free, but when able, a small payment is expected | *In- and Out-Surgeons*, Mr. Lennox Browne, M, Th. 2.30; Dr. Orwin, W. S. 2.30; Dr. Grant, Tu. 5, Th. 2.30; Messrs. P. S. Jakins, M. W. 2.30; F. 5; T. Carmalt Jones, M. Th. S. 2.30 |
| Charing Cross Hospital, West Strand, W.C. | *In- and Out-Surgeon*, Mr. Cantlie, F. 9 |
| Great Northern Central Hospital, Caledonian Rd, N. | *In- and Out-Surgeon*, Mr. A. E. Cumberbatch, W. 9 |
| Guy's Hospital, St. Thomas's Street, S.E. | *In- and Out-Surgeon*, Mr. Purves, Tu. F. 12.30 |
| Hospital for Diseases of the Throat, Golden Square, W. Sec., Mr. W. T. Sharp | *Physicians*, Drs. Wolfenden, Tu. F. 2.30; Macdonald, Tu. F. 6.30; Bond, W. S. 2.30 *Surgeon*, Mr. T. M. Hovell, M. Th. 2.30 |

### EAR CASES—*Continued.*

| Hospitals and Terms of Admission. | Physicians, Surgeons, and Medical Officers, with Days and Hours of Attendance. |
|---|---|
| King's College Hospital, Portugal Street, W.C. | *In- and Out- Surgeon*, Mr. Pritchard. Th. 2.30 |
| London Hospital, Whitechapel Road, E. | *In- and Out-Surgeons*, Dr. Woakes, Mr. T. M. Hovell, S. 9 |
| Middlesex Hospital, Berners Street, W. Free. | *Out-Surgeon*, Mr. Hensman, Tu. 9 |
| National Hosp. for the Paralysed, etc., Queen Sq., W.C. | *In- and Out-Surgeon*, Mr. A. E. Cumberbatch, W. 2 |
| North West London Hospital, 18, Kentish Town Rd. | *Out-Surgeon*, Mr. Collier, M. 1.30 |
| St. Bartholomew's Hospital, Smithfield, E.C. | *Surgeon*, Mr. Cumberbatch, Tu. F. 2 |
| St. George's Hospital, Hyde Park Corner, S.W. | *In- and Out-Surgeon*, Sir W. B. Dalby, Tu. 1.30 |
| St. Mary's Hospital, Cambridge Place, Paddington, W. | *In- and Out-Surgeon*, Mr. G. P. Field, W. S. 9.30 |
| St. Thomas's Hospital, Westminster Bridge Road, S.E. | *Out-Surgeon*, Mr. Clutton, M. 1.30 |
| University College Hospital, Gower Street, W.C. | *In- and Out-Surgeon*, Mr. Barker, Th. 9 |
| Westminster Hospital, Broad Sanctuary, S.W. | *In- and Out-Surgeon*, Mr. Barrow, Tu. F. 9 |

### EYE DISEASES.

| | |
|---|---|
| Central London Ophthalmic Hosp., 238A, Gray's Inn Rd., W.C. Sec., Mr. G. H. Leah. Admission free | *Surgeons*, Messrs. T. Archer, G. Abbott, W. Charnley, W. Jessop, E. Clarke, J. R. Kemp and Granville Barker (House Surgeon). Daily, 1 |
| Evelina Hosp. for Sick Children, Southwark Bridge Rd. | *Surgeon*, Dr. W. Brailey, Th. 1 |
| Great Northern Central Hospital, Caledonian Rd., N. | *Surgeon*, Mr. Hutchinson, Jnr., Tu. F. 9.30 |
| Guy's Hospital, St. Thomas's Street, S.E. | *Surgeons*, Mr. Higgens, Dr. Brailey, Tu. F. 12.30 |
| Hospital for Sick Children, 49, Gt. Ormond Street, W.C. | *Surgeon*, Mr. R. Marcus Gunn, Tu. 2 |
| King's College Hospital, Portugal Street, W.C. | *Surgeon*, Mr. McHardy, M. W. Th. 1.30 |
| London Hospital, Whitechapel Road, E. | *Surgeons*, Messrs. Waren Tay, S. 9; Eve, Tu. 9 |
| Middlesex Hospital, Berners Street, W. | *Surgeon*, Mr. Lang, Tu. F. 9 |
| National Hosp. for the Paralysed, etc. Queen Sq., W.C. | *Surgeons*, Messrs. R. Brudenell Carter, F. 4; R. Marcus Gunn, Tu. 4 |
| North-West London Hosp. 18, Kentish Town Rd., N.W. | *Surgeon*, Dr. W. Collins, F. 3.30 |
| Paddington Green Children's Hosp., Paddington Grn. | *Surgeon*, Mr. W. H. H. Jessop, Th. 9 |
| Royal Free Hospital, Gray's Inn Road, W.C. | *Surgeon*, Mr. Mackinlay, M. F. 9 |
| Royal London Ophthalmic Hospital, Blomfield Street, Moorfields, E.C. Sec., Mr. R. J. Newstead. Admission free to the poor. | *Surgeons*, Messrs. J. W. Hulke, W. S.; G. Lawson, Tu. F.; J. Couper, Tu. F.; W. Tay, M. Th.; J. Tweedy, Tu. F.; E. Nettleship, W. S.; R. M. Gunn, W. S.; Lang, M. Th. Hours, 8 to 10 |
| Royal South London Ophthalmic Hosp., 6, St. George's Circus, S.E. Sec., Mr. C. Comyn. Free to the necessitous poor; or by payment | *Surgeons*, Messrs. Watson, McHardy. Daily, 2 |
| Royal Westminster Ophthalmic Hospital, King William Street, W.C. Sec., Mr. T. Beattie-Campbell. Poor and accidents free | *In- and Out-Surgeons*, Messrs. Power, M. F.; Frost, M. Th.; Rouse, Tu. S.; Hartridge, Tu. F.; Macnamara, M. Th.; Cowell, W. S.; Juler, W. S. Hour, 1 |
| St. Bartholomew's Hospital, Smithfield, E.C. | *Surgeons*, Messrs. Power, Th. 2; Vernon, Tu. S. 2.30 |
| St. George's Hospital, Hyde Park Corner, S.W. | *Surgeons*, Messrs. B. Carter, Frost, M. Th. 2; F. 1.15; W. S. 2 |
| St. Mary's Hospital, Cambridge Place, Paddington. W. | *Surgeons*, Messrs. A. Critchett, H. Juler, Tu. F. 9.30 |
| St. Thomas's Hospital, Westminster Bridge Road, S.E. | *Surgeons*, Messrs. Nettleship, Tu. Th. F. 1.30; Lawford, M. W. 1.30 |
| University College Hospital, Gower Street, W.C. | *Surgeon*, Mr. Tweedy, M. Tu. Th. F. 2 |
| West London Hospital, Hammersmith Road, W. | *Surgeons*, Messrs. B. Vernon, H. Dunn, Tu. Th. S. 2 |
| Westminster Hospital, Broad Sanctuary, S.W. | *Surgeon*, Mr. Cowell, M. Th. 2 |
| Western Ophthalmic Hosp. 153 and 155, Marylebone Rd., W. Sec., Mr. G. E. Manton. By letter or small payments; free to poor | *Surgeons*, Drs. Miller, M.; Collins, F.; Wheatly, M. Th.; Messrs. Carmal, Jones, W.; Buxton, Tu. F.; Nunn W. S. Hour, 2 |

